# The Longitudinal Associations of Body Dissatisfaction with Health and Wellness Behaviors in Midlife and Older Women

**DOI:** 10.3390/ijerph20247143

**Published:** 2023-12-05

**Authors:** Lisa Smith Kilpela, Savannah C. Hooper, Casey L. Straud, Victoria B. Marshall, Christina L. Verzijl, Tiffany M. Stewart, Taylur T. Loera, Carolyn Black Becker

**Affiliations:** 1 Department of Medicine, UT Health San Antonio, San Antonio, TX 78229, USA; 2 ReACH Center, UT Health San Antonio, San Antonio, TX 78229, USAtloer01@jaguar.tamu.edu (T.T.L.); 3 Barshop Institute, UT Health San Antonio, San Antonio, TX 78229, USA; 4 Audie Murphy Veterans Hospital, South Texas VA Health System, San Antonio, TX 78229, USA; 5 Department of Psychiatry and Behavioral Sciences, UT Health San Antonio, San Antonio, TX 78229, USA; 6 Department of Adolescent Medicine, Dell Children’s Medical Group, University of Texas at Austin, Austin, TX 78712, USA; 7 Pennington Biomedical Research Center, Louisiana State University, Baton Rouge, LA 70803, USA; tiffany.stewart@pbrc.edu; 8 Department of Psychology, Texas A&M University San Antonio, San Antonio, TX 78224, USA; 9 Department of Psychology, Trinity University, San Antonio, TX 78212, USA; cbecker@trinity.edu

**Keywords:** body dissatisfaction, mental health, midlife, older women, wellness, quality of life

## Abstract

Emerging research suggests that body dissatisfaction (BD) is prevalent among midlife and older women (i.e., upwards of 70%). Cross-sectionally, BD is associated with myriad poor health and wellness outcomes (e.g., depression, disordered eating, bad nutrition) in midlife/older women. However, relatively few studies have examined the longitudinal relations between BD and health outcomes in this population. This preliminary study investigated the longitudinal associations of BD with wellbeing and health-related quality of life (QOL) among midlife/older adult women over one year. Participants (*n* = 86, women aged 40–72 years, *M* = 51.49, SD = 7.34, 86% white) completed self-report measures of BD, psychosocial impairment, health behaviors, and QOL at baseline (T1) and 12-month follow-up (T2). A series of multiple linear regression models included T1 BD as the predictor variable of health outcomes at T2, covarying for T1 BMI and age in all models. BD was associated with greater negative emotions and psychosocial impairment, less physical activity enjoyment, and poorer physical, psychological, and social QOL one year later. Findings suggest that BD is associated with negative consequences for women across the lifespan (ƒ^2^ ranges = 0.06–0.60). Future research investigating BD as a unique, modifiable risk factor for health outcomes among diverse samples of midlife/older women is warranted. Targeting BD in interventions may improve health indices beyond eating disorders for this population.

## 1. Introduction

Body dissatisfaction (BD) is a well-established risk factor for numerous adverse health outcomes and behaviors among young women and girls. For instance, in addition to eating disorders [1], BD is associated with depression, lower levels of physical activity and consumption of nutrient-dense foods, unhealthy weight control behaviors, smoking, and reduced health-related quality of life (QOL) in younger populations (e.g., [2,3,4]). In adolescents, BD also prospectively predicts thin ideal internalization, eating disorder symptoms, self-esteem, suicidal ideation, and depression (e.g., [5,6,7,8,9]).

Although historically investigated among younger female samples, emerging research suggests that BD remains prevalent among midlife (ages 40–59) and older (ages 60+) women (e.g., [10,11,12,13]). For instance, Runfola et al. (2013) found that 88.9% of women aged 45–54 experienced BD; 89% of women aged 55–64, 88.2% of women aged 65–74, and 71.9% of women ages 75+ also reported BD [14]. Yet, little is known regarding the health (both physical and psychological) and wellness correlates of BD in midlife/older women, especially the longitudinal associations. Even less is known about the extent to which BD acts as a longitudinal risk factor for poor physical and psychological health outcomes in these same populations. Indeed, the existing research examining health correlates of BD in midlife/older female samples is both preliminary and limited in its cross-sectional nature (e.g., [15]).

Based on longitudinal data from younger populations (e.g., [2,3,4,5,6,7,8,9]), BD has been identified as a nonspecific risk factor for a myriad of negative physical and mental health outcomes. This information has informed the development of interventions aimed at reducing body dissatisfaction and its sequelae in younger ages (e.g., [16]). However, the paucity of literature examining how BD is longitudinally associated with health outcomes among age-diverse samples means that the field lacks the needed justification for intervention development. Moreover, such prospective data in age-diverse samples might elucidate risk factor interactions for negative health indices in older populations, which would necessitate modifications of interventions developed for younger-aged groups. Finally, without longitudinal data to investigate potential prospective associations in age-diverse samples, the field lacks a nuanced understanding of how BD may interact differently across different developmental life stages.

With respect to the existing BD research in older women, early cross-sectional data indicate that BD is associated with depression, dieting behaviors, eating disorder symptoms, thin ideal internalization, and greater engagement in negative age-related body talk in midlife/older women (e.g., [12,17,18,19]). More recent data suggest that BD is associated with psychosocial impairment, lower diet quality, depression, and anxiety in this subpopulation (e.g., [15,20,21,22]). Fewer studies have investigated the association between BD and health behaviors, wellness, or QOL [23]. Mond et al. (2013) examined the association of adult women’s BD with QOL in a sample of 5255 Australian women aged 18 to 42 years [24]. The results indicated that mental health QOL, psychosocial functioning, and some aspects of physical health functioning all decreased as BD increased. BD was found to be cross-sectionally associated with negative affect (i.e., experiencing a variety of negative emotions or emotional distress), worse sleep, decreased consumption of nutritious foods, greater psychosocial impairment, and worsened health-related QOL in a sample of women aged 50–86 [20]. Many associations remained when controlling for negative affect.

Of note, none of these cross-sectional relations have been examined longitudinally in midlife/older women. Considering that BD both longitudinally predicts negative health outcomes in adolescents and cross-sectionally relates to poorer physical/psychological health indices in midlife/older women, it may be the case that BD is longitudinally associated with negative outcomes for midlife/older women. If this supposition were to hold, that would further support the case for increased longitudinal research in this area and, potentially, the development of body image interventions designed for midlife/older women and the needed financial support for testing these interventions.

Importantly, some evidence suggests that the associations between BD and wellbeing indices may decline as women age. Some studies indicate that physical appearance becomes less important with age among midlife/older women. For instance, Rodgers et al. (2016; [25]) examined women’s reactions to physical changes associated with middle-age via responses on a Facebook thread answering the question “Does the voice in your head get kinder as you get older?” Results found that 56% of midlife women responded with an affirmative comment that this voice gets kinder with age, suggesting a decrease in the importance of physical appearance [25]. Thus, if appearance becomes less important with age, BD may have an attenuated impact on wellbeing in midlife versus in younger adulthood. Another study among women aged 25–86 found that those women aged 61+ years had the highest percentage of participants endorsing extreme body dissatisfaction [20]. Thus, investigation into the potential longitudinal relations between BD and both physical and psychological health outcomes among midlife/older adult women is warranted.

Ultimately, investigating longitudinal associations of BD in ages beyond young adulthood will move us one step closer to understanding whether BD may impact the wellbeing of midlife/older adult women. Therefore, the current study sought to begin to address a gap in the literature via a longitudinal evaluation of health and wellness correlates (physical and psychological) of BD over the course of one year in midlife/older women. The primary aims of this study were to examine the cross-sectional and longitudinal associations of BD in midlife/older women (aged 40+) with key psychological (e.g., negative mood and psychosocial impairment), physical health (frequency of consuming nutritious foods, enjoyment of physical activity), and health-related QOL indicators over the course of one year.

We hypothesized that greater BD would be cross-sectionally associated with worse psychosocial health, physical health, and health-related QOL outcomes, regardless of participant age, at baseline. We further hypothesized that greater baseline BD would be longitudinally associated with psychological health, physical health, and health-related QOL outcomes at one-year follow-up, regardless of age.

## 2. Methods

### 2.1. Participants

Participants (*n* = 86) were women aged 40–72 years (*M* = 51.49, SD = 7.34), predominantly non-Hispanic white (86%), and married/cohabitating (77.9%); BMI ranged from 16.76–44.62 k/m^2^. This sample was highly educated, with 80.2% reporting a bachelor’s degree or higher (see Table 1).

### 2.2. Procedure

Adult women aged 40 years and older who were English-speaking, U.S. residents, and able to consent to participation were recruited via public-facing materials online (e.g., digital flyers, social media postings from organizations with vested interests in health, women’s issues, or aging) or print flyers at local organizations/businesses. Potential participants either self-selected by viewing recruitment materials or received materials through snowball sampling via social media sites (e.g., Facebook, Twitter), personal and professional networks through email (e.g., colleagues in the body image field), postings within local organizations (e.g., local YMCAs) via online platforms, or word of mouth. Recruitment materials described the study as exploring wellness and body image in adult women and requested that women forward the study invitation to their own networks (i.e., snowball sampling) either digitally by sharing materials on social media, email, or web postings or by sharing the print flyer with their networks of women.

After consenting to participate in the study, participants completed self-report questionnaires online. Participants completed questionnaires at baseline (T1) and 12-month follow-ups (T2). Upon completion of the questionnaires at each time point, participants could provide email addresses (primary and secondary) to enter a lottery for a USD 200 Amazon gift card. Only responses from eligible participants that passed validity checks (e.g., checks for unreasonably short completion time, invalid text, repeated entries, response biases indicating inattention or not reading) were included in analyses.

Participants who provided their email were automatically contacted for a follow-up survey 12 months later. A total of 334 women completed the survey at T1 and 86 completed the survey at T2 (25.7% retention). We conducted one-way ANOVAs to compare demographics and outcomes of non-completers (only completed T1) and completers (completed T1 and T2). The results revealed no significant differences in age, BMI, or any measure scores at T1 between completers and non-completers. Based on spontaneous participant feedback from non-completers, the primary stated reason for attrition was length and time-intensity of the survey, and the option to enter the raffle appeared to be an insufficient incentive for participant retention in this demographic. Due to longitudinal analyses and concerns with imputing too much data, we conducted analyses with the subsample that completed both time points of the study (*n* = 86) (see Table 2 for T1 and T2 descriptives).

### 2.3. Measures

Regarding demographics, participants self-reported age, race, ethnicity, highest level of education, and marital status.

#### 2.3.1. Body Dissatisfaction

The Body Shape Questionnaire (BSQ; [26]) assessed BD; we used the 8-item version (BSQ-8D; [27]). The BSQ-8D uses a 6-point Likert scale (1 = Never, 6 = Always) with items summed for a total score; higher scores indicate greater BD. Existing short-form BSQ scores can be categorized into degrees of BD: no concern  =  <19, mild concern  =  19–25; moderate concern  =  26–33; marked concern  = >33). The BSQ-8D has strong reliability and validity, and the shortened versions have similar convergent and discriminant validity [27,28]. Two questions were replaced from the BSQ-8D (e.g., “Have you imagined cutting off fleshy areas of your body?”) to target less pathological body concerns as we were concerned that this item was too graphic for an older adult audience. Internal consistency for the modified BSQ-8D sample was excellent at baseline (Cronbach’s α = 0.92), indicating that the construct held with this modified version.

#### 2.3.2. Psychological Health

We assessed negative affect with the 17-item negative affect subscale of the Positive and Negative Affect Schedule (PANAS; [29,30]) comprising the fear, guilt, and sadness subscales. Items are rated on a 5-point Likert scale (1 = Very slightly or not at all, 5 = Extremely); higher total scores indicate greater experience of negative emotions, with good construct validity [30]. Internal consistency was high in the current sample (α = 0.97).

To measure psychosocial impairment due to body image, we used the 16-item Clinical Impairment Assessment Questionnaire (CIA-Q; [31]). The CIA-Q assesses impairment in four domains of life (mood/self-perception, cognitive functioning, interpersonal functioning, and work performance) secondary to eating, exercise, and weight/shape concerns over the past 28 days. Items were on a 4-point Likert scale (0 = Not at all, 3 = A lot). Scores are summed; higher scores indicate greater psychosocial impairment. Research supports CIA-Q internal consistency and construct validity [31]. Because our primary focus of this study was body image, we modified the CIA-Q instructions to omit mentions of eating habits and exercise. Thus, participants only completed the CIA-Q in relation to weight and shape in order to isolate participants’ perceived impact of BD on psychosocial functioning (versus confounding the potential effects of eating or exercise concerns). The modified version demonstrated high internal consistency (α = 0.96), indicating that the construct held in this modified version.

#### 2.3.3. Health and Wellness Behaviors

The 2-item Eating Behaviors Questionnaire (EBQ; [24]) measured the consumption of nutrient-dense foods. Items were rated on a 5-point Likert scale (1 = Consume at every meal, 5 = Never). The items are summed and higher scores indicate fewer attempts to eat nutritionally dense foods (α = 0.73).

The 8-item Physical Activity Enjoyment Scale (PACES; [32]) assessed positive affect associated with physical activity. Items were rated on a 5-point Likert scale (1 = Disagree a lot, 5 = Agree a lot) with items summed for total scores. Higher scores indicate greater physical activity enjoyment. We chose PACES because positive affect associated with physical activity is both a predictor of engagement in physical activity as well as an outcome of it in older adults. Additionally, we chose this briefer measure (8 items versus the Physical Activity Scale for the Elderly [33], which includes 12 core items with secondary sub-items, totaling 24 items) to be mindful of participant burden. Internal consistency in this sample was high (α = 0.96).

#### 2.3.4. Quality of Life (QOL)

We used the World Health Organization QOL Scale (WHOQOL-BREF; [34] to measure health-related QOL. This version has 26 items that measure overall QOL over the last 4 weeks within four domains: physical health (e.g., activities of living), psychological health (e.g., self-esteem), social relationships (e.g., social support), and environment (e.g., financial resources). Items are scored on a 5-point Likert scale, with subscales summed for a raw score. Raw scores are then transformed by subtracting the lowest possible raw score and dividing it by possible raw score range. That number is then multiplied by 100 to obtain the final score. Total scores range from 0 to 100; higher scores indicate better QOL. Research supports the internal consistency of the subscales [35]. Internal consistency in this sample was good (α: physical health = 0.81, psychological = 0.89, environment = 0.82, social relationships = 0.76).

### 2.4. Data Analytic Plan

Inspection of the data included Q–Q plots to evaluate the normality of the dependent variables and variance inflation factor (VIF) and tolerance statistics to assess concerns for multicollinearity. An examination of Q–Q plots indicated that data met the assumption of normality to proceed with parametric tests (i.e., linear regression models). VIFs (range = 1.013–1.046) and tolerance (range = 0.956–0.987) for all models hung around 1.00, indicating no concerns for multicollinearity.

Overall, the mean BD in this sample was moderate at baseline and mild at follow-up (Table 2). At T1, only 19.8% of participants reported no BD, per BSQ cutoffs (i.e., BSQ < 19 indicates no concerns); 25.6% reported mild BD, 20.9% reported moderate BD, and 33.7% reported marked BD. At T2, 36.0% reported no concerns, 29.1% reported mild BD, 16.3% reported moderate BD, and 18.6% reported marked BD.

Correlations between T1 and T2 variables are presented in Table 2. A series of linear regression models were used to address the study aims. Cross-sectional relations were examined using regression models that included the continuous variable T1 BD as the predictor (x) of health/wellness outcomes at T1 (y); the same analyses were conducted for longitudinal associations, but with health/wellness outcomes at T2 (y). We covaried for T1 BMI and T1 age in all models; BMI was calculated using self-reported weight and height. Consistent with prior literature in this field, we included BMI as a covariate as it has been linked with BD. We also covaried for age, based on our hypotheses. Effect sizes (Cohen’s ƒ^2^) for BD were calculated using the unique unadjusted variance (R^2^) attributed by BD in each model [36]. Of note, because of the small sample size and high correlations between T1 and T2 outcomes (Table 2), we were unable to control for time in the longitudinal analyses to examine BD as a unique predictor of negative health/wellness outcomes. Therefore, this study is preliminary and only looks at longitudinal associations, not predictions.

## 3. Results

### 3.1. Cross-Sectional Associations

Table 3 presents the cross-sectional associations at baseline. Higher T1 BD was cross-sectionally associated with greater psychosocial impairment (variance = 37.6%, ƒ^2^ = 0.60, *p* < 0.001), higher negative affect (variance = 32%, ƒ^2^ = 0.47, *p* < 0.001), less enjoyment of physical activity (variance = 9.1%, ƒ^2^ = 0.10, *p* = 0.003), and worse QOL (physical [variance = 14.4%, ƒ^2^ = 0.17, *p* < 0.001], psychological [variance = 32.2%, ƒ^2^ = 0.47, *p* < 0.001], and social [variance = 18.4%, ƒ^2^ = 0.22, *p* < 0.001]); greater BD was associated with poorer health outcomes. Nutritious food consumption and environmental QOL were nonsignificant. Regarding covariates, higher BMI was associated with worse nutrition (variance = 9.9%, *p* = 0.005); lower BMI (variance = 9.8%, *p* = 0.006) and older age (variance = 4.6%, *p* = 0.05) were associated with greater physical activity enjoyment. Older age was associated with better psychological QOL (variance = 4.8%, *p* = 0.04).

### 3.2. Longitudinal Associations

Table 4 presents the longitudinal association of baseline (T1) BD with T2 outcomes. Higher T1 BD was longitudinally associated at T2 with greater psychosocial impairment (variance = 31.1%, ƒ^2^ = 0.45, *p* < 0.001), higher negative affect (variance = 19.2%, ƒ^2^ =0.23, *p* < 0.001), less physical activity enjoyment (variance = 9.3%, ƒ^2^ = 0.10, *p* = 0.003), and worse QOL (physical [variance = 6.1%, ƒ^2^ = 0.06, *p* = 0.03], psychological [variance = 25.8%, ƒ^2^ = 0.35, *p* < 0.001], and social [variance = 5.5%, ƒ^2^ = 0.06, *p* = 0.03]). Nutritious food consumption and environmental QOL were nonsignificant. Regarding covariates, older age was associated with less negative affect (variance = 7.4%, *p* = 0.01). Higher BMI (variance = 8.9%, *p* = 0.005) and younger age (variance = 6.1%, *p* = 0.02) were associated with worse nutrition. BMI (variance = 9.8%, *p* = 0.006) and older age (variance = 4.6%, *p* = 0.05) were associated with greater enjoyment of physical activity, while older age was associated with better psychological QOL (variance = 6.3%, *p* = 0.02).

## 4. Discussion

The eating disorders and body image fields have been plagued by the eating disorders stereotype (i.e., thin, young, affluent, white), resulting in a majority of research being conducted with young, non-Hispanic white, female samples [37,38]. This study sought to expand the literature by increasing age-diversity in the field by examining associations of an established eating disorders risk factor, BD, with psychological and physical health outcomes in midlife/older adult women. We want to acknowledge upfront that more work is needed in the eating disorders field and the related body image field to improve the inclusion of individuals from diverse ethnic, racial, gender, sexual orientation, and other minoritized communities [37,38] in order to better elucidate both the unique and overlapping considerations for working with diverse individuals with disordered eating and/or body dissatisfaction. While the sample in the current study improves age diversity in the field by focusing on a midlife/older population, this sample lacks racial/ethnic diversity (i.e., 86% non-Hispanic white, which is consistent with prior research in the field [37,38]). Thus, we want to both extend and amplify the call for more research with underrepresented populations in the eating disorder and body image fields. This study comprises an early step in this process by delving into the longitudinal associations of BD with psychological and physical health/wellness indices in a previously underreported population of midlife and older adult women.

Although recent research in the eating disorders and body image fields has examined body image in midlife/older women, relatively few studies have examined the longitudinal relations between BD and psychological health, health/wellness behaviors, and QOL in women beyond adolescence or young adulthood. Those that have examined the relation of BD to health outcomes in age-diverse samples (e.g., [39,40]) are limited in their scope of health outcomes (e.g., only psychological wellbeing and self-esteem). While one study found that body shame and body esteem, two facets of body image that are related but distinct from BD, were correlated with psychological wellbeing in midlife women [39], another study [40] found that BD was not related to self-esteem in women over 30 years of age. Therefore, additional research is needed to determine whether BD is a modifiable, prospective risk factor for mental health outcomes among age-diverse women. The current study takes the first step of examining the relations between BD and health/wellness outcomes both cross-sectionally and at one-year follow-up in a sample of midlife/older adult women. Our hypotheses that greater BD would be associated with poorer health/wellness outcomes both cross-sectionally and one year later, regardless of age, were largely supported.

Higher BD was both cross-sectionally and longitudinally related to greater negative affect, psychosocial impairment, less enjoyment of physical activity, and poorer psychological, social, and physical QOL. Effect sizes for BD (both cross-sectionally and longitudinally) were largest for psychosocial constructs, suggesting that the greatest magnitude of association is with psychological health. Importantly, the effect size for BD on enjoyment of physical activity was medium; positive affect associated with engaging in physical activity is of particular relevance for older populations [32]. Thus, BD may play an important role in both psychosocial health and health behaviors. These findings are consistent with recent cross-sectional data suggesting that BD is correlated with poorer wellness in adult women. In younger women (aged 18–42 years), the strongest cross-sectional correlations of BD have been within the domains of mental health and psychosocial functioning [24], which is similar to our findings in midlife/older women. Furthermore, in samples of adult women across the lifespan, BD was cross-sectionally associated with negative affect (e.g., [20,41]). In midlife women (aged 40–45), greater BD was cross-sectionally correlated with greater psychological distress [42], which is consistent with our findings.

Regarding health-related QOL, our findings extend those of past cross-sectional research indicating that greater BD is associated with poorer QOL. For instance, psychosocial impairment related to body image and disordered eating was cross-sectionally correlated with BD [20] and body image disturbance [43] in adult women across the lifespan. Additionally, previous research found that BD was cross-sectionally associated with health and emotional QOL in a sample of midlife (aged 40–65) women [44]. Thus, our findings align with previous research in this population, suggesting that these negative associations of BD hold in midlife/older women. Our results also extend prior research on cross-sectional correlations into longitudinal associations.

Contrary to our hypothesis, nutritional intake and environmental QOL were not related to BD one year later, despite cross-sectional associations between BD and nutrition in age-diverse samples (e.g., [20,22]). In contrast, previous research has found that BD prospectively predicts nutritional intake in younger samples (e.g., [3,45]). Thus, our preliminary data in midlife/older women suggest that this longitudinal association may not extend into later life. Further research engaging larger samples of midlife/older women is needed to better understand if, how, or why this may be the case.

Furthermore, BMI was nonsignificant in the vast majority of models, indicating that BD has a significant relationship with wellness indices across midlife/older adulthood regardless of BMI status. This finding indicates that BD is a concern for women across the weight spectrum and that interventions optimally should be developed that appeal to women with both lower- and higher-weight bodies. Our findings also suggest that interventions targeting BD, as opposed to BMI, might be more important for improving health, wellness, and QOL in midlife/older women. In fact, efforts focusing on BMI and weight loss will likely increase BD [46] among women, thus resulting in iatrogenic harm to health, mental health, and QOL among aging populations. Importantly, our findings are consistent with a growing body of literature highlighting the importance of emphasizing non-BMI or weight-related interpretations of health status [46,47] or intervention approaches. Data are mounting on the harmful link between body dissatisfaction and various health indices (e.g., [24]). Thus, more efforts are needed in our field to advocate for the importance of research and resources to improve BD as a conduit to improving health and wellness.

It is important to note that we only examined longitudinal associations without accounting for time. Because of the small sample size and strong correlations between T1 and T2 health/wellness outcomes in the current sample (Table 2), we lacked the statistical power to examine BD as a unique, prospective risk factor for poor health/wellness outcomes by controlling for baseline levels of health/wellness variables. As such, we were unable to determine the causality of BD on health/wellness outcomes. It is possible that these constructs have bidirectional relations, such that health indices may influence BD and BD may influence health status. Thus, future research examining the potential unique, prospective influence of BD on various health and wellness outcomes among midlife/older women will be an important next step in understanding how BD affects this population.

Specifically, determining if BD is a risk factor in midlife/older women would have significant implications. In younger populations, there is robust evidence that BD can be changed through effective interventions [48], indicating that BD is a modifiable risk factor. If BD is a modifiable risk factor for health/wellness indices in midlife/older adult women, reducing BD may be an important method of increasing wellbeing and QOL in this demographic, thus making a broader impact on the overall wellbeing of women in midlife and beyond. Notably, interventions aimed at improving BD in midlife/older populations likely need to be age-tailored to best meet the needs of this population. Thus, future research is needed to investigate age-tailored interventions for BD in midlife/older populations (e.g., to investigate targeting BD as a method of increasing wellbeing and QOL in midlife/older populations [49,50]) and explore potential differences in the consequences of BD within this demographic. While most research to date has shown the impact of intervening in BD to decrease ED pathology [16], it is possible that targeting BD will have a broader impact on the overall wellbeing of women in midlife and beyond. Overall, more research is needed to establish if BD is indeed a relevant modifiable risk factor in the health of midlife/older women. Ultimately, this will move the field beyond longitudinal associations and toward prospective predictive studies adequately powered to examine the unique variance of BD among age-diverse populations in order to best inform future intervention efforts.

While these findings are novel in this population and potentially carry significant implications, this study has limitations. Although a strength of this study is the longitudinal design, retention was low (25.7%). The sample size was also small and lacked racial/ethnic diversity as the majority of participants identified as non-Hispanic white. While this is consistent with samples in the eating disorders field, this represents an important limitation to the generalizability of these findings to populations outside of the US, to diverse and minoritized groups of midlife/older women, and to older men. Future research examining intersectional disadvantages in eating disorder health disparities, such as older adults from historically minoritized communities, is needed. Additionally, sampling online may have contributed to the low retention rate at follow-up and narrow sampling characteristics, which also could have biased results. The small sample size represents the preliminary nature of these data in the literature and limits the implications and generalizations that can be drawn from our findings.

Furthermore, because this study only included women, this work does not contribute to understanding body image in men. Although the literature on body image and eating disorders in men is growing, more research is needed to identify common and unique themes in the experiences of body dissatisfaction in relation to health outcomes in men. It is important to replicate the findings of this study in a larger, more ethnically and economically diverse sample of midlife/older women, using more comprehensive or in-person assessment methods.

Furthermore, the EBQ and BSQ are narrow in their assessment of each construct. The EBQ is a noncomprehensive assessment of nutritional intake; the BSQ only examines one dimension of body image (i.e., BD). Future research should explore other dimensions of body image and a detailed examination of nutritional intake in this age population. Finally, this sample had a broad age range across midlife/older women; however, we controlled for age in all models. Future research should explore potential differences in the various consequences of BD between midlife and older women to investigate potential nuances of BD experiences and consequences in various developmental life stages.

## 5. Conclusions

In sum, results from the current study suggest that BD is cross-sectionally and longitudinally associated with poorer wellbeing and health-related QOL in midlife/older women and that BMI is largely unrelated to indices of poorer wellbeing and health-related QOL one year later. Although previous literature has shown that individuals place a greater emphasis on health and body function with age [51], BD retains a significant relation with the health, wellness, and QOL of women in midlife/older adulthood. Overall, the results highlight the need for more research examining whether BD is a modifiable risk factor in this population. The identification of modifiable, prospective risk factors that impede healthy aging is integral in efforts to prolong health span (i.e., the duration of life in good health). Ultimately, BD may be an important intervention target for improving health, wellbeing, and QOL in women as they age, which could improve support for women’s health across the lifespan.

If future research supports BD as a risk factor for poorer wellbeing and health-related QOL in midlife/older women, then a critical next step will be to develop targeted interventions to improve BD in this population. Currently, many healthcare providers focus on reducing BMI in those living in larger bodies. This research suggests that it may be more impactful to focus on BD and move from weight-centric healthcare, which may increase BD, to a weight-inclusive paradigm.

## Figures and Tables

**Table 1:** Sample demographics at T1 (*n* = 86).

Demographics	*M* (SD) or *n* (%)
Age	51.49 (7.34)
Body Mass Index	26.74 (6.48)
Race/Ethnicity	
White	74 (86%)
Hispanic/Latina	3 (3.5%)
Native Hawaiian or Other Pacific Islander	1 (1.2%)
Multiple Races or “Other”	8 (9.3%)
Education	
High School Graduate	6 (7%)
Some College	11 (12.8%)
Bachelor’s Degree	22 (25.8%)
Some Graduate School	5 (5.8%)
Graduate Degree	42 (48.8%)

**Table 2:** Baseline and 12-month follow-up descriptives and correlations.

	Baseline (T1)		12-Month Follow-Up (T2)		T1-T2 Correlations
Variables	*M*	SD	*M*	SD	
BSQ ^a§^	27.94	10.36	23.91	9.64	0.78
PANAS ^§^	32.63	17.04	29.76	14.73	0.81
CIAQ ^§^	9.74	12.02	7.95	10.99	0.86
EBQ ^§^	3.99	1.79	3.65	1.45	0.67
PACES ^¥^	32.76	6.64	33.15	7.08	0.68
BMI	26.74	6.48	26.67	6.47	0.97
QOL ^¥^					
Physical	78.82	16.78	80.99	13.18	0.75
Psychological	67.97	21.99	70.83	19.30	0.86
Social	67.73	23.07	67.54	23.33	0.77
Environmental	81.69	14.97	84.60	12.99	0.77

Note. BSQ = Body Satisfaction Questionnaire; PANAS = Positive and Negative Affect Schedule; CIAQ = Clinical Impairment Assessment Questionnaire; EBQ = Eating Behaviors Questionnaire; PACES = Physical Activity Enjoyment Scale; BMI = body mass index; QOL = quality of life. ^a^ There was a significant difference (*p* < 0.001) between T1 and T2. ^§^ Higher scores indicate poorer health. ^¥^ Higher scores indicate better health.

**Table 3:** Cross-sectional linear regression outcomes with T1 BD as the predictor variable of outcomes at T1.

Variables	b	[95% CI]	β	*t* Value	BD R^2^	Adj. R^2^	BD ƒ^2^
PANAS ^§^	0.97	[0.66, 1.27]	0.58	6.29 ***	0.32	0.34	0.47
CIAQ ^§^	0.74	[0.53, 0.94]	0.62	7.09 ***	0.38	0.39	0.60
EBQ ^§^	0.02	[−0.02, 0.05]	0.09	0.87	0.01	0.09	0.01
PACES ^¥^	−0.20	[−0.33, −0.07]	−0.31	−3.07 **	0.09	0.21	0.10
QOL ^¥^							
Physical	−0.63	[−0.97, −0.29]	−0.39	−3.69 ***	0.14	0.14	0.17
Psychological	−1.25	[−1.63, −0.86]	−0.58	−6.41 ***	0.32	0.36	0.47
Social	−0.98	[−1.44, −0.52]	−0.44	−4.27 ***	0.18	0.17	0.22
Environmental	−0.30	[−0.62, 0.02]	−0.20	−1.87	0.04	0.07	0.04

Note. BMI and age were included as covariates in all models. PANAS = Positive and Negative Affect Schedule; CIAQ = Clinical Impairment Assessment Questionnaire; EBQ = Eating Behaviors Questionnaire; PACES = Physical Activity Enjoyment Scale; BMI = body mass index; QOL = quality of life; BD R^2^ = variance accounted for by BD in each model; Adj. R^2^ = adjusted R^2^ for full model, including covariates; BD ƒ^2^ = effect size for BD variance within each model. ƒ^2^ effect size interpretation: 0.02 = small, 0.15 = medium, 0.35 = large; ** *p* < 0.01, *** *p* < 0.001. ^§^ Higher scores indicate poorer health. ^¥^ Higher scores indicate better health.

**Table 4:** Linear regression outcomes with T1 BD as the predictor variable of outcomes at T2.

Variables	b	[95% CI]	β	*t* Value	BD R^2^	Adj. R^2^	BD ƒ^2^
PANAS ^§^	0.64	[0.36, 0.92]	0.45	4.55 ***	0.19	0.24	0.23
CIAQ ^§^	0.61	[0.41, 0.81]	0.57	6.19 ***	0.31	0.34	0.45
EBQ ^§^	0.02	[−0.01, 0.05]	0.13	1.20	0.02	0.13	0.02
PACES ^¥^	−0.21	[−0.34, −0.07]	−0.31	−3.09 **	0.09	0.20	0.10
QOL ^¥^							
Physical	−0.32	[−0.61, −0.04]	−0.25	−2.25 *	0.06	0.04	0.06
Psychological	−0.98	[−1.34, −0.62]	−0.52	−5.45 ***	0.26	0.30	0.35
Social	−0.55	[−1.05, −0.04]	−0.24	−2.16 *	0.06	0.03	0.06
Environmental	−0.19	[−0.48, 0.09]	−0.15	−1.36	0.02	0.03	0.02

Note. BMI and age were included as covariates in all models. PANAS = Positive and Negative Affect Schedule; CIAQ = Clinical Impairment Assessment Questionnaire; EBQ = Eating Behaviors Questionnaire; PACES = Physical Activity Enjoyment Scale; BMI = body mass index; QOL = quality of life; BD R^2^ = variance accounted for by BD in each model; Adj. R^2^ = adjusted R^2^ for full model, including covariates; BD ƒ^2^ = effect size for BD variance within each model. ƒ^2^ effect size interpretation: 0.02 = small, 0.15 = medium, 0.35 = large; * *p* < 0.05, ** *p* < 0.01, *** *p* < 0.001. ^§^ Higher scores indicate poorer health. ^¥^ Higher scores indicate better health.

## Data Availability

The data presented in this study are available upon request from the first author (Lisa Smith Kilpela; kilpela@uthscsa.edu).
